# Preferences and Expectations for Home Robot Tasks: Comparison According to Age and Household Type in Republic of Korea

**DOI:** 10.3390/bs14111070

**Published:** 2024-11-08

**Authors:** Ha-Yeon Yoo, Minjun Kim

**Affiliations:** 1 College of Art & Design, Ewha Womans University, Seoul 03760, Republic of Korea; hyoo@ewha.ac.kr; 2 Department of Industrial Engineering, Ajou University, Suwon 16499, Republic of Korea

**Keywords:** home robot task, willingness to use, social status of home robot, comparison by age group, comparison by household type

## Abstract

Most studies of consumer preferences and expectations for home robots focus on either older adults or single-person households (SPHs). However, with the rise in voluntary SPHs among young adults and seniors, it is critical to compare both age and household types in robot research. This study explored perceptions of home robots and willingness to use their features based on age and household type, in the context of the expanding home robot market in Republic of Korea. An online survey of 400 individuals was conducted, targeting young SPHs and multi-person households (MPHs) in their 20s and 30s as well as older SPHs and MPHs in their 50s and 60s. The survey covered four robot task categories, with 40 items derived from previous research: household chores (20 items), personal care (seven items), leisure/companion (nine items), and health (four items). The results helped predict the main target groups for each in-home robot task by identifying items that showed differences in responses between groups and interpreting these based on age, household type, and their combination. The study provides valuable data on consumer expectations, highlighting differences in responses according to both age and household type, offering insights for the robotics industry to effectively target their products.

## 1. Introduction

Robots have been the subject of active research and development on the technology side [1]. These are now making inroads into the lives of ordinary humans in industrial environments and in the form of various home robots. In recent years, home robots have been highlighted as a product category to be aware of at the Consumer Electronics Show [2]. Familiar consumer electronics brands such as LG, Samsung, and Panasonic, as well as Amazon and Dyson, have accelerated their robot product development. Startups also continue to introduce companion home robots [3,4]. Home robots are also referred to as consumer robots. These are defined by the Institute of Electrical and Electronics Engineers (IEEE) as “robots you can buy and use just for fun or to help you with tasks and chores” [5]. These are distinguished from industrial robots and are categorized further into edutainment, security, collaborative (chore), and personal assistance robots according to their main functions [6]. Although service robots are increasingly common in the marketplace, much of the existing research has focused on applications in the retail and hospitality sectors (e.g., [7,8]), with relatively little attention paid to their use in home environments. In fact, research on home robots accounts for only 6.98% of the total literature on robot adoption ([9]).

Collaborative and personal assistance robots, as part of the home robot category, are particularly relevant to the increase in single-person households (SPHs) and an aging population. Although several studies have investigated user expectations and task preferences for home robots, many have focused on robots as a means of assisting elderly or mobility-impaired patients with activities at home [10,11,12,13,14,15,16]. However, when we consider the growth of SPHs in demographics, we observe this across all age groups rather than only among older individuals. The SPH is the fastest-growing type of household in many regions of the world owing to the variations in institutional arrangements, demographic behaviors, and labor migration in the past few decades. Widowed individuals and many young and middle-aged adults who are divorced or have never married live alone. The number of married couples living apart is also increasing and constitutes a growing share of those living alone [17]. This trend is global: In England (2020), Republic of Korea (2020), France (2021), and Japan (2020), the share of SPHs exceeded 30% of the total number of households. In Germany (2020), Sweden (2021), and Finland (2021), it exceeded 40% of the total number of households [18]. Voluntary SPHs have increased among young individuals in their 20s and 30s and among older ones in their 50s and 60s. “Voluntary SPHs” means SPHs by personal choice [19], or they are single because they want to be so [20]. The reasons for the increase in voluntary SPHs include independence from family by having a job in younger age groups, declining interest in long-term relationships and marriage, changing perceptions of divorce, and maintaining independent living after the death of a spouse in older age [19,20,21].

As discussed above, notwithstanding the increase in SPHs extending to all age groups, including younger individuals, many studies on the perceptions and task preferences of home robots have either focused on older adults or have compared older and younger age groups. For example, Scopelliti et al. [22] examined the differences between young, middle-aged, and older adults in their attitudes toward technology, assessment of robot capability, and emotional reactions to a home robot. Ezer [23] evaluated the age differences by surveying younger and older adults with regard to their expectations of home robots and their impact on robot acceptance. Biswas et al. [14] compared the perspectives of older and younger individuals on multimodal human–robot interfaces. Research has also considered factors such as users’ possessiveness toward robots and technology acceptance tendencies, such as early adopters and mass users. For example, Park and del Pobil [24] examined attitudes toward service robots in Republic of Korea and found that the “need to belong” moderately influenced users’ beliefs about robots. Similarly, Saari et al. [25] examined the acceptance of social robots, highlighting that increasing perceived usefulness through practical benefits and user-friendly design significantly increases adoption. They also found that early adopters prioritize the demonstrability of outcomes, while mass market users place more value on perceived enjoyment.

Notwithstanding the importance of examining both household type and age in conjunction, few studies have considered both age differences and household type variations. Park and Ryoo [26] studied the preferences for home robot care services by categorizing respondents into eight household types: single males, single females, and various combinations with children. Although the household type was considered in their study, it differed from the research on robot tasks by household type because it focused on care service targets rather than home robot tasks. Understanding home robots from the perspective of a wider range of tasks—such as household chores, personal care, leisure and companionship, and healthcare—is crucial for their successful integration into domestic settings. These tasks reflect the diverse needs of households and directly influence the willingness to use these robots.

The characteristics of household members, such as age and the number of household members, affect the types of relationships within the household and the pool of economic resources [27], which ultimately affects lifestyle and consumption within the household. For example, the inclusion of older people in MPHs affects household priorities and decision-making, such as healthcare consumption and the potential contribution to the household budget from pensions [28]. In addition, while the rise in the number of older SPHs is a major contributor to the dramatic increase in SPHs, the recent rise in younger SPHs is also a major factor. While these are the same household type, they have different lifestyles and different needs for home robots due to the age difference, and thus, it was necessary to analyze both age and household type in combination.

To address this trend, this study was motivated by the following question: “Do differences by age group and household type exist?” That is, the study aimed to evaluate the main tasks of home robots that Korean consumers wish to apply and the role (status) of robots in homes by age group and household type. According to Statistics Korea, “In 2021, SPHs accounted for 33.4% of the total households. This is expected to reach 39.6% by 2050 in Republic of Korea” [18]. Republic of Korea is an attractive target because it has a highly steep SPH growth trend, is culturally receptive to technology and new products, and is an active leader in new trends. These make it a testbed that can be translated into a global phenomenon on a smaller scale. On the home robot front, sales of home robots such as cleaning robots, education robots, and pet robots are growing rapidly in Korean households because individuals are spending more time at home after COVID-19 [29].

This study examined and categorized the main tasks of home robots based on a literature review and investigated the differences in consumer responses by age group, household type, and combination of age and household type using an online survey of 400 individuals in Republic of Korea. Thus, the study formulated the following basic hypotheses and examined the degree of willingness to use home robots by task among Korean consumers:

**H1.**  *There is no difference in willingness to use robot task items by age.*

**H2.**  *There is no difference in willingness to use robot task items by household type.*

**H3.**  *There is no difference in willingness to use robot task items according to the combination of age and household type.*

This study contributes to the literature by defining and categorizing a task list of home robots based on previous studies and ‘analyzing how users’ willingness to use these tasks on the list and their perceptions of the robot’s role in the home differ by age and/or household type.

Home robots are highly connected to the broader ecosystem of smart home technologies [30,31]. Home robots will play a central role in the implementation and maintenance of smart home systems. These systems will efficiently monitor user activity, understand context from multiple data sources, intervene through actuators or robots, interact with users, provide companionship, and notify medical personnel when necessary [32]. For such systems to function effectively, the seamless integration of various IoT devices, such as smartphones, in-home cameras, and voice assistants (e.g., Amazon Alexa, Google Assistant), is essential. This interconnectedness allows the system to automatically provide context-aware functionality or suggest useful actions based on the user’s needs. In this environment, home robots would play a crucial role, especially in providing services and interacting with users, thus forming the core of a truly smart and responsive home environment. By exploring users’ willingness to adopt home robots, particularly in terms of task relevance and household composition, our study provides fundamental insights into how future innovations in smart device technology can meet the diverse needs of modern households. These findings underscore the importance of our research in advancing both home robotics and the broader field of smart home technologies, highlighting how robots can act as central hubs within these interconnected systems.

The remainder of this paper is organized as follows: In Section 2, the literature on home robots is reviewed to identify the tasks of home robots. In addition, this section presents the groundwork and methods for conducting online surveys. Section 3 presents the results of the online survey. It highlights items that differed significantly according to age and/or household type. Section 4 discusses the design implications of the results, and Section 5 summarizes the results and suggests future research issues.

## 2. Related Works and Methods

### 2.1. Main Tasks for Home Robots

Considering previous studies to define the main tasks for home robots, Ezer [23] examined the main tasks that young adults and older adults imagine for robots in the home in order to understand the potential of domestic robots. They categorized the tasks into cleaning/chores, security, physical assistance, other computer tasks, cooking, maintenance/repair, service, entertainment, health, and companionship/conversation. The study determined that the most imagined tasks across all age groups are important functions such as cleaning/chores, security, and physical assistance. Meanwhile, tasks with high interaction with the robot (such as entertainment and companion/conversation) or those in which the robot forms a friendship with the user were not favored. Older individuals were more willing than younger ones to use a robot for vital monitoring or help in an emergency. Smarr et al. [13] investigated the preferred robot tasks in homes among the elderly in the U.S. Based on the participants’ brainstorming, they divided the 48 tasks into six categories: personal care, leisure activities, health, chores, information management, and object manipulation. According to the results, the respondents preferred to use robots for household chores, manipulating objects, and information management. This was because these tasks are tedious, the robot can perform these better, the respondents require help with a task that they are not good at, and they prefer human assistance to robots for tasks related to personal care and leisure activities. Ajaykumar et al. [11] conducted an extensive interview-based survey of older adults with limited mobility regarding tasks for which they would prefer robotic assistance. The tasks that the respondents answered were grouped into five categories: household, care, companion, cognition, and safety. In addition, these respondents preferred physical assistance with household tasks (particularly those considered tedious, hazardous, or difficult, e.g., bed-making, surface cleaning, moving objects, laundry, and ironing) and emergency response over social/care assistance. This is in line with the convenience, efficiency, comfort, reliability, and well-being cited as the potential advantages of robots. However, they do not desire assistance with high-level decision-making or tasks with personal implications (e.g., feeding a dog) because they do not want robots to intervene in their social interactions.

**Figure 1:**
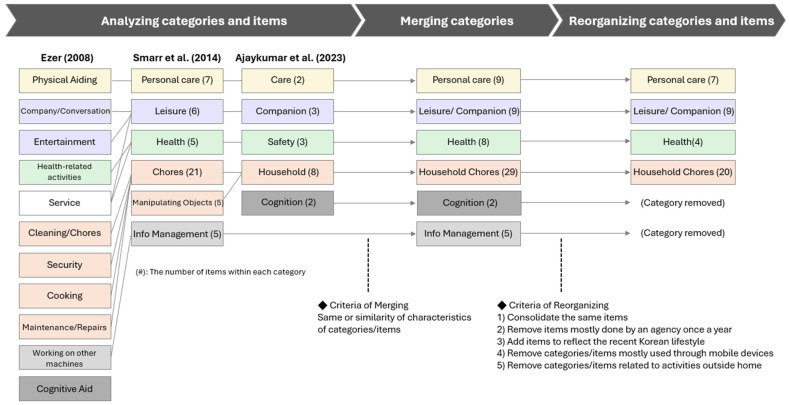
Defining task categories from previous studies [11,13,23].

Based on the aforementioned studies, the main tasks of home robots can be categorized into four main categories: “household chores”, “personal care”, “leisure/companion”, and “health”. These task categories were derived through the following process (Figure 1): First, all the categories and items of robot tasks defined in the studies by Ezer [23], Smarr et al. [13], and Ajaykumar et al. [11] were listed and compared. Then, the characteristics of each category/item were analyzed and consolidated into similar categories. Finally, the task categories and items for this study were reorganized with the following criteria: (1) consolidating the same items, (2) removing services that use professional agencies about once a year in Republic of Korea, (3) adding pet-related items to reflect the recent lifestyle trend [33], (4) removing categories/items that are mainly used through mobile devices, and (5) removing categories/items related to activities outside the home.

Detailed task items have been defined for each category as listed in Table 1. Specifically, “household chores” includes 20 items that a robot can assist with at home, “personal care” comprises seven basic daily items necessary for daily life, “leisure/companion” covers nine items related to learning and social activities, and “health” includes four items addressing health and emergency situations.

### 2.2. Online Survey

To investigate the differences in perceptions of home robots by age group and/or household type among potential users and consumers, the age groups were young individuals in their 20s and 30s and older individuals in their 50s and 60s, and the household types were SPHs and multi-person households (MPHs). An online survey was conducted by Research & Yu, a specialized survey company in Seoul, Republic of Korea, to ensure a uniform distribution of the target group; that is, the users were categorized into four groups: young SPHs, young MPHs, older SPHs, and older MPHs. One hundred individuals were recruited in each group, i.e., a total of 400 individuals. The online survey was conducted in October 2023, and individual consent was obtained prior to the start of the survey (Appendix A).

The main survey items were questions regarding the willingness to use the previously defined home robot task categories and items under each category. Each question was answered on a 5-point Likert scale (not at all willing to use, not willing to use, moderately willing to use, highly willing to use, extremely willing to use). The “willingness to use” question was designed to determine the willingness and desire to use (which is related to preference) considering the respondent’s living environment and conditions realistically. The respondents were then asked in a multiple-choice format why they preferred a home robot. They were encouraged to answer freely if they did not have the desired answer. Finally, the respondents were asked to provide their opinions on the desired role of a home robot in a multiple-choice format. They were given the option of providing additional comments if they did not have the desired answer. This was to understand the consumers’ perceptions of home robots. To conclude, the total number of questions was 46: 4 on the willingness to use categories, 40 on the willingness to use specific task items, and 2 on the reasons for preferring robots and the role of robots in the home.

One limitation of relying solely on surveys is that respondents may only answer pre-defined questions, potentially missing other relevant insights. To mitigate this, we considered text mining as a complementary method. Text mining allows valuable insights to be extracted from unstructured data such as user reviews, providing a richer, more nuanced understanding of user preferences and experiences. However, due to the currently limited variety of available home robots (e.g., primarily robotic vacuum cleaners and AI speakers), there is insufficient data to perform effective text mining analysis. Furthermore, since our study focuses on users’ future willingness to use more advanced home robots, text mining of existing product reviews would not capture users’ intentions to adopt future technologies. Therefore, we chose to use a structured survey approach because it provided a more direct way to capture users’ perceptions and intentions regarding the broader range of home robot tasks. Nevertheless, future research could incorporate text mining as a complementary approach as the home robot market continues to expand.

In addition, the Likert scale was chosen because of its wide acceptance for measuring attitudes and subjective experiences, particularly in studies examining user acceptance and perceived usefulness of technology. Its simplicity allows participants to easily express their level of agreement or disagreement, which is particularly important for capturing nuanced perceptions across multiple domains, such as household chores, healthcare, and personal care tasks. While alternative methods such as semantic differential scales or ranking tasks were considered, we chose the Likert scale because of its reliability, ease of interpretation, and alignment with previous technology acceptance studies, ensuring consistency and comparability with existing literature (e.g., [34,35]).

## 3. Results

The online survey results were analyzed to assess differences in responses based on (1) age group, (2) household type, and (3) combinations of age and household type. For age group analysis (1), responses from 200 young and 200 older participants were compared. For household type analysis (2), responses from 200 SPHs and 200 MPHs were compared. For the combined analysis (3), four groups of 100 respondents each were compared: young SPHs, young MPHs, older SPHs, and older MPHs. A *t*-test was used for analyses (1) and (2), while a two-way analysis of variance (ANOVA) was employed for analysis (3) to determine statistically significant differences between groups.

### 3.1. Results for Task Categories

The results of the willingness to use robots for each task are as follows: The overall average willingness scores across the four task categories were 3.98 for “Household chores”, 3.92 for “Health”, 3.56 for “Personal care”, and 3.46 for “Leisure/companion”. As shown in the bold item in Table 2, the willingness to use scores for the “household chores” category differed significantly between the younger group (average score of 3.90) and the older group (average score of 4.07). Meanwhile, none of the other task categories showed significant differences between the groups by age, household, and combinations of age and household type. This implies that among the four categories, “household chores” showed the highest overall intention to use, and the elderly showed a higher intention to use than younger individuals. This is similar to the results of previous studies such as Smarr et al. [13].

### 3.2. Results for Items in Each Task Category

Table 3 shows the results of the willingness to use robots for the 40 task items. According to the results of “Household chores” items, the following items showed significant differences between the groups: the items that differed by age group were ‘Grocery shop’, ‘Change light bulbs’, ‘Clean bedrooms’, ‘Clean floors’, ‘Clean kitchen’, ‘Wash pet’, and ‘Manipulating objects’. The elderly are more likely than younger individuals to use the robots for basic daily tasks that require frequent cleaning cycles and more cumbersome tasks such as water cleaning (particularly in cleanliness-sensitive areas such as kitchens, bedrooms, and floors) and that can help with age-related physical limitations, such as manipulating objects. This is consistent with the low willingness to use among older adults compared with younger adults for activities that require relatively long cycles, such as changing light bulbs. ‘Grocery shop’ is more likely to be used by younger individuals than older ones. This may be because younger individuals are more accustomed to using mobile application-based services rather than performing grocery shopping in person; therefore, they are more comfortable with having a robot perform it for them.

Meanwhile, ‘Prepare meals’ showed the only difference between household types within the “Household chores” category, regardless of age group. SPHs were more likely to use these than MPHs. This could be because busy SPHs find it more cumbersome to prepare food for themselves or because the act of preparing food for other family members is associated with social interaction between family members, and they do not desire for a robot to be involved in this task (when referring to Ajaykumar et al. [11]). When comparing both household type and age group, significant differences were found for ‘Clean bedrooms’, ‘Clean floors’, and ‘Wash pet’. ‘Wash pet’ involved a higher willingness to use among young SPHs (particularly compared with older SPHs). This is related to the recent trend wherein young SPHs in Republic of Korea are living away from their families for study or work and relying on their pets. While ‘Walk with pet’ and ‘Feed pet’ did not show differences between groups, they exhibited statistically significant interaction effects, with the highest willingness to use found among young SPHs, followed by older MPHs. This could be interpreted as an indication that the respondents’ families in the older group included younger members of the household or that even if they were only in the older age group, they were in an MPH situation with many other household tasks and required robotic assistance with daily pet care tasks directly related to hygiene.

The survey analysis of the items under the “Personal care” category revealed significant differences in the following items: the items that differed by age group were ‘Shave’, ‘Walk’, and ‘Brushing teeth’. Older people are more likely than younger people to use items that help them overcome physical limitations, such as walking aids. Meanwhile, items such as ‘Shave’ and ‘Brushing teeth’ are more likely to be used by younger individuals. This may be because older individuals prefer to have a human rather than a robot perform these tasks. This is in line with the results of Smarr et al. [13]. In the case of ‘Wash/comb hair’ and ‘Bathe’ in the “personal care” category, young SPHs have the highest willingness to use, followed by older MPHs, older SPHs, and young MPHs. This is difficult to interpret as a difference by age group or household type. However, it can be interpreted that the young SPH group requires a robot to minimize time and effort (even for tasks performed on their own body) if they need to perform it every day.

The survey analysis of the task items under the “Leisure/companion” category revealed significant differences in the following items: ‘Being entertained’, ‘Call family/friends’, ‘Learn to use new technology’, ‘Get information on hobbies’, ‘Reading stories’, and ‘Conversation’. It is generally assumed that robots for leisure and companionship in the home would be preferred by younger age groups and that the need would be higher in SPHs where interaction between household members is not feasible. However, it is noteworthy that the young SPH group scored the lowest for ‘Learn to use new technology’ and that older individuals were more likely to use robots than younger ones for several tasks, including ‘Being entertained’, ‘Call family/friends’, ‘Learn to use new technology’, ‘Obtain information on hobbies’, ‘Reading stories’, and ‘Conversation’. This difference in responses across age groups could be attributed more to older individuals’ need to conveniently learn new things and obtain information on their own without the aid of others and their desire to talk to another individual (perhaps anyone including robots) in lonely situations, rather than their familiarity with robots. The relatively low distribution of willingness to use scores for ‘Entertain guest’ compared with the other tasks indicates a lower preference for robotic intervention in human interaction. This is similar to the observations of Ajaykumar et al. [11] and Smarr et al. [13]. This may also be related to the decline in the culture of entertaining guests at home in Korean society.

The survey analysis of the task items under the “Health” category found no statistically significant differences between groups based on age, household type, or their combination. Although willingness to use the items was moderately higher among older SPHs as expected, these differences were not statistically significant. All groups have higher robot preferences for dealing with life-threatening emergencies and medication reminders, which are considered instrumental tasks [36]. This can be interpreted as people prefer the robot’s ability to be a tool to help them in situations rather than their health itself. However, regarding tasks that affect their physical health, such as prescribing medication, robots are less favored by all groups. This can be interpreted as many people are uncomfortable with the idea of relying on new technologies such as robots or AI to diagnose illnesses and recommend treatments, as well as concerns about the security of their health records [37].

### 3.3. Respondents’ Perceptions of Robot

Table 4 shows the survey results on the reasons why respondents like home robots. ‘Saving time’ was the most common reason for willing to use a home robot (33.7%), followed by ‘Saving effort’ (28.6%), ‘Overcoming physical challenges’ (16.0%), ‘Confidence in capabilities’ (12.9%), ‘Social features’ (8.4%), and ‘Others’ (0.3%). For all the respondent groups, ‘Saving time’ was the most common, followed by ‘saving effort’.

Table 5 indicates the survey results for the expected role of home robots. ‘Secretary/Butler’ was the most common role for the home robot (36.3%), followed by ‘Tools (appliances)’ with 32.2%, ‘Friend’ with 16.0%, ‘Family member’ with 9.1%, and ‘Pet’ with 6.5%. ‘Secretary/Butler’ ranked first for young MPHs, older SPHs, and older MPHs; however, ‘Tools (appliances)’ ranked first for young SPHs. This revealed differences between the groups.

## 4. Discussion: Design Implications for Home Robots

In Section 3, a statistical analysis of the survey results and an interpretation of the reasons for the differences in responses by age and household type are presented. Table 6 presents the outcome of mapping the results of the analysis in Section 3 to the list defined in Table 1. At the category level, “Household chores” showed significant differences by age group. Among the 40 individual task items, 16 showed significant differences by age group, four showed significant differences by household type, and 12 showed differences between the age and household type comparison groups. Therefore, the hypotheses “There is no difference in willingness to use robot task items by age (H1)”, “There is no difference in willingness to use robot task items by household type (H2)”, and “There is no difference in willingness to use robot task items by age and household type (H3)” were rejected by some task items. This implies that household robot tasks vary by task category and task items according to the consumer age group and/or household type.

The results indicate that research and product development for home robots that perform specific functions may benefit from targeting consumer groups that are primarily age-based, focus on household type, or a combination of both. For example, robot product manufacturers have an advantage in designing for the elderly rather than the young in the ‘Household chores’ category. In particular, in the “Household chores” category, robots that help clean bedrooms, floors, and kitchens, which need to be kept clean on a daily basis, are highly preferred by older people, so it is important to consider the usability of older people when designing robot products, such as incorporating age-friendly interactions (simple and clear user guides, easy ways to control functions, etc.). In the case of grocery shopping, it is effective to design services and interfaces that take into account the trends of young people. For robot companies that provide functions such as preparing meals, it is recommended to develop robot interfaces that consider the usability of all ages. However, since single-person households are preferred to multi-person households, it will be advantageous to have a robot that performs functions optimized for single-person meal preparation and has a compact size and movement that does not take up much space in the home. For pet care-related functions, it is important to target young SPHs and design a robot that can safely interact with pets while considering their preferences. Conversely, a more cautious approach and higher focus on outreach may be required when targeting groups that are less likely to use a robot to perform a specific task.

The results of the “Personal care” category can be used to predict key targets by individual task items. For example, shaving and brushing teeth are best targeted at young consumers (particularly those in SPHs), and washing/combing hair is best targeted at SPHs rather than MPHs. However, for robots performing walking assistance tasks, it is important to target older users, particularly those living alone.

Although the individual task items in the “Leisure/companion” category show a low intent to use compared with the other categories, the insight is that targeting by age is important for most tasks (such as being entertained, calling family/friends, learning to use new technology, obtaining information on hobbies, reading stories, and conversation). These tasks can be assisted in the comfort of the home for older adults who may have difficulty learning new things and are more likely to become disconnected from conversations. Among them, being entertained, learning to use new technology, getting information on hobbies, and conversation, it is necessary to conduct research and development that simultaneously considers the differences in household types of the elderly.

Even if factors such as age or household type do not directly influence the willingness to use home robots for the “Health” category, it is crucial to prioritize data privacy. As emphasized by Koczkodaj et al. [38], the consequences of health-related data breaches are serious and raise significant concerns. Therefore, when designing such robots, data privacy issues need to be carefully considered, particularly with regard to how robots manage sensitive data in the home. This includes addressing how personal and health data of household members will be securely stored and shared to ensure that users’ privacy is protected in all interactions with the robot.

Saving time, saving effort, and overcoming physical difficulties are the factors generating positive perceptions of home robots. These are related to the strong preference for “Household chores” items that require repetitive actions. This directly supports the interpretation that the task items favored by young individuals are related to comfort and convenience. The response ‘able to overcome physical difficulties’ is related to the result wherein the older group prefers to perform frequent household tasks and tasks involving walking and movement. Ultimately, these observations are consistent with the fact that Korean robot users consider home robots as assistants (i.e., secretary/butler) who help humans with household activities, rather than as family members or friends. In particular, young SPHs tend to consider robots as ‘Tool (appliance)’. This supports the conclusion that they constitute the group that seeks convenience and efficiency from robots the most.

## 5. Conclusions

This study investigated the tasks of home robots that consumers are willing to use and the roles they expect robots to play by age and household type (which are the main factors that determine the actual consumer and consumer environments in which robots are used) at a time when home robot products are penetrating Republic of Korea. Previous studies focused on identifying preferred robot tasks by age group or the elderly, whereas this study analyzed the differences in willingness to use home robots and perceptions of home robot tasks by age and/or household type. Overall, the results of this study are similar to those of previous studies [11,13,16] in that the elderly showed higher intentions to use them for repetitive and cumbersome household chores tasks, lower intentions to use them for companion/social tasks, and more positive responses to the emergency response task. However, this study observed differences in the detailed task items by age group, household type, or both. This indicates that the type of household in which robots coexist may also be an important consideration. For example, younger SPHs are more likely to actively pursue efficiency and perceive robots as home appliances. This is different from the case with the other groups. Younger SPHs, in addition to elderly SPHs, may be important independent research groups for home robots.

This study contributes to marketing research based on data collection and analysis from a “consumer” perspective through its survey of potential customers to investigate their perceptions and willingness to use home robots. It also provides strategic insights for the industry, such as which robotic products to focus on based on consumer age and household type, or what age and household type of consumer is effective to target when designing robots to perform specific tasks. However, this study has research limitations such as limiting the survey to people living in Korea, a small sample size, and a quantitative survey method that does not provide in-depth insights into the specific reasons for the responses and the causes of interactions, so follow-up research is needed to compensate for these limitations. In other words, it is necessary to expand the geographical coverage to other countries with increasing SPHs and increase the sample size. In addition, it is necessary to reorganize robot task items based on AI considering changes in robotics trends, and to conduct qualitative research such as interviews to explore specific reasons for the willingness to use each task.

## Figures and Tables

**Table 1:** Task categories and item list.

Household Chores (20)	Personal Care (7)	Leisure/Companion (9)	Health (4)
Prepare meals (HC1)	Shave (PC1)	Entertain guests (LC1)	Decision for medication (HT1)
Set table (HC2)	Wash/comb hair (PC2)	Being entertained (LC2)	Remind to take medicine (HT2)
Grocery shop (HC3)	Bathe (PC3)	Call family/friends (LC3)	Exercise (HT3)
Wash dishes by hand (HC4)	Eat/feed myself (PC4)	Learn to use new technology (LC4)	Call doctors/911 (HT4)
Clean refrigerator (HC5)	Get dressed (PC5)	Get information on hobbies (LC5)	
Laundry (HC6)	Walk (PC6)	Learn new skills (LC6)	
Water plants (HC7)	Brushing teeth (PC7)	Exercising together (LC7)	
Sort mail (HC8)		Reading stories (LC8)	
Use dishwasher (HC9)		Conversation (LC9)	
Take out trash/recyclables (HC10)			
Make bed/change sheets (HC11)			
Change light bulbs (HC12)			
Clean bedrooms (HC13)			
Clean windows (HC14)			
Clean floors (HC15)			
Clean kitchen (HC16)			
Walk with pet (HC17)			
Feed pet (HC18)			
Wash pet (HC19)			
Manipulating objects (HC20)			

**Table 2:** Comparison of the results of average willingness to use score for each task category.

Task Categories	Age			Household Type			Combination of Age and Household Type				
	Young (n = 200)	Older (n = 200)	*p*-Value ^a^	SPHs (n = 200)	MHPs (n = 200)	*p*-Value ^b^	Young SPHs (n = 100)	Young MPHs (n = 100)	Older SPHs (n = 100)	Older MPHs (n = 100)	*p*-Value ^c^
**Household chores**	3.90	4.07	**0.05**	4.00	3.96	0.73	3.89	3.9	4.10	4.03	0.23
Personal care	3.58	3.54	0.66	3.58	3.54	0.58	3.63	3.53	3.54	3.54	0.85
Leisure/ companion	3.4	3.52	0.23	3.46	3.45	0.88	3.44	3.36	3.49	3.54	0.59
Health	3.88	3.96	0.28	3.96	3.88	0.34	3.91	3.84	4.01	3.92	0.56

Note. The bold and shaded category indicates a statistically significant difference in any of the comparisons between age, household type, or their combinations, where the *p*-value is 0.1 or below. ^a^ Comparing results by age group (i.e., young vs. older) using the *t*-test. ^b^ Comparing results by household type (i.e., SPHs vs. MPHs) using the *t*-test. ^c^ Comparing results by combinations of age and household type (i.e., four groups) using two-way ANOVA.

**Table 3:** Comparison of the results of average willingness to use score for all task items.

Tasks	Items	Age Groups			Household Types			Combinations of Age Groups and Household Types				
		Young (n = 200)	Older (n = 200)	*p*-Value ^a^	SPHs (n = 200)	MHPs (n = 200)	*p*-Value ^b^	Young SPHs (n = 100)	Young MPHs (n = 100)	Older SPHs (n = 100)	Older MPHs (n = 100)	*p*-Value ^c^
Household chores	**Prepare meals**	3.58	3.50	0.39	3.62	3.46	**0.10**	3.89	3.9	4.1	4.03	0.32
	Set table	3.34	3.29	0.64	3.35	3.27	0.46	3.63	3.53	3.54	3.54	0.86
	**Grocery shop**	3.54	3.31	**0.02**	3.41	3.44	0.81	3.44	3.36	3.49	3.54	0.15
	Wash dishes by hand	3.69	3.62	0.46	3.69	3.62	0.58	3.91	3.84	4.01	3.92	0.73
	Clean refrigerator	3.41	3.4	0.96	3.46	3.36	0.35	3.67	3.49	3.57	3.42	0.80
	Laundry	4.01	4.00	0.88	4.01	4.00	0.88	3.38	3.3	3.33	3.25	0.98
	Water plants	3.50	3.60	0.36	3.56	3.55	0.96	3.52	3.56	3.3	3.31	0.83
	Sort mail	3.36	3.26	0.33	3.35	3.27	0.49	3.76	3.63	3.61	3.62	0.31
	Use dishwasher	3.81	3.83	0.85	3.90	3.75	0.17	3.44	3.38	3.47	3.34	0.59
	Take out trash/recyclables	3.96	3.86	0.29	3.89	3.93	0.67	4.00	4.02	4.02	3.97	0.64
	Make bed/change sheets	3.66	3.60	0.54	3.65	3.62	0.76	3.52	3.49	3.59	3.61	0.90
	**Change light bulbs**	3.60	3.39	**0.06**	3.54	3.46	0.48	3.47	3.25	3.22	3.3	0.27
	**Clean bedrooms**	3.77	4.04	**0.00**	3.88	3.94	0.50	3.88	3.75	3.91	3.76	**0.03**
	Clean windows	4.06	4.15	0.34	4.03	4.18	0.11	3.97	3.95	3.81	3.91	0.31
	**Clean floors**	4.22	4.42	**0.01**	4.29	4.35	0.49	3.69	3.63	3.6	3.6	**0.09**
	**Clean kitchen**	3.94	4.14	**0.03**	4.06	4.03	0.71	3.64	3.56	3.43	3.35	0.17
	Walk with pet	3.10	3.07	0.80	3.07	3.1	0.80	3.75	3.79	4.00	4.09	0.21 *
	Feed pet	3.46	3.43	0.76	3.41	3.48	0.52	4.00	4.12	4.06	4.24	0.24 *
	**Wash pet**	3.25	3.02	**0.06**	3.17	3.11	0.64	4.20	4.25	4.39	4.45	**0.02**
	**Manipulating objects**	3.92	4.08	**0.09**	3.94	4.06	0.21	3.95	3.93	4.17	4.12	0.18
Personal care	**Shave**	2.77	2.50	**0.01**	2.68	2.6	0.47	3.21	2.99	2.93	3.21	**0.05**
	**Wash/comb hair**	3.19	3.14	0.69	3.27	3.06	**0.05**	3.54	3.39	3.28	3.58	0.01 *
	**Bathe**	3.06	2.97	0.45	3.1	2.93	0.15	3.42	3.08	2.91	3.14	0.10 *
	Eat/feed myself	3.41	3.23	0.11	3.36	3.27	0.46	3.90	3.95	3.99	4.17	0.34
	Get dressed	3.00	2.88	0.26	3.00	2.87	0.22	2.88	2.67	2.48	2.53	0.21
	**Walk**	3.06	3.58	**0.00**	3.37	3.27	0.39	3.44	2.93	3.10	3.18	**0.00**
	**Brushing teeth**	3.04	2.86	**0.10**	3.06	2.85	**0.06**	3.26	2.86	2.94	3.00	**0.08**
Leisure/ companion	Entertain guests	3.31	3.33	0.76	3.35	3.29	0.48	3.48	3.34	3.24	3.21	0.81
	**Being entertained**	3.54	3.77	**0.01**	3.64	3.67	0.71	3.14	2.86	2.87	2.88	**0.10**
	**Call family/friends**	3.42	3.6	**0.08**	3.51	3.51	1.00	3.11	3.01	3.62	3.53	0.29
	**Learn to use new technology**	3.85	4.08	**0.01**	3.88	4.05	**0.06**	3.19	2.90	2.92	2.80	0.00 *
	**Get information on hobbies**	3.89	4.14	**0.00**	4.00	4.04	0.65	3.37	3.24	3.34	3.33	**0.04**
	Learn new skills	3.71	3.73	0.84	3.73	3.71	0.76	3.53	3.55	3.75	3.8	0.97
	Exercising together	3.81	3.80	0.96	3.81	3.8	0.96	3.46	3.38	3.56	3.64	0.98
	**Reading stories**	3.54	3.71	**0.07**	3.56	3.69	0.14	3.66	4.05	4.11	4.05	0.12
	**Conversation**	3.58	3.92	**0.00**	3.81	3.7	0.28	3.86	3.92	4.13	4.15	**0.01**
Health	Decision for medication	3.52	3.51	0.88	3.52	3.52	0.96	3.71	3.71	3.76	3.70	0.97
	Remind to take medicine	4.17	4.29	0.15	4.17	4.29	0.15	3.83	3.78	3.78	3.82	0.19
	Exercise	3.81	3.85	0.74	3.83	3.83	0.91	3.5	3.58	3.61	3.81	0.98
	Call doctors/911	4.33	4.42	0.26	4.41	4.36	0.53	3.65	3.52	3.97	3.88	0.53

Note. Bold and shaded items indicate a statistically significant difference in any of the comparisons between age, household type, or their combination, where the *p*-value is 0.1 or below. ^a^ Comparing results by age groups (i.e., young vs. older) using the *t*-test. ^b^ Comparing results by household types (i.e., SPHs vs. MPHs) using the *t*-test. ^c^ Comparing results by combinations of age and household type (i.e., four groups) using two-way ANOVA. * Statistically significant interaction effects between age groups and household types.

**Table 4:** Reasons why they are fond of home robots.

Reason		Overcoming Physical Challenges	Saving Time	Saving Effort	Confidence in Capabilities	Social Features	Others
Total	F *	150	316	268	121	79	3
	%	16.0	33.7	28.6	12.9	8.4	0.3
Young SPHs	F	38	75	61	33	16	1
	%	17.0	33.5	27.2	14.7	7.1	0.4
Young MPHs	F	33	78	65	31	14	1
	%	14.9	35.1	29.3	14.0	6.3	0.5
Older SPHs	F	40	78	64	29	19	1
	%	17.3	33.8	27.7	12.6	8.2	0.4
Older MPHs	F	39	85	78	28	30	0
	%	15.0	32.7	30.0	10.8	11.5	0.0

* F: frequency of multiple choice responses.

**Table 5:** Expected roles of home robots.

Roles		Tools (Appliances)	Secretary /Butler	Friend	Family Member	Pet
Total	F *	213	240	106	60	43
	%	32.2	36.3	16.0	9.1	6.5
Young SPHs	F	61	53	30	14	10
	%	36.3	31.5	17.9	8.3	6.0
Young MPHs	F	50	60	19	13	12
	%	32.5	39.0	12.3	8.4	7.8
Older SPHs	F	48	64	25	15	11
	%	29.4	39.3	15.3	9.2	6.7
Older MPHs	F	54	63	32	18	10
	%	30.5	35.6	18.1	10.2	5.6

* F: frequency of multiple choice responses.

**Table 6:** Mapping results of the task item list.

Household Chores	Factors			Personal Care	Factors			Leisure /Companion	Factors			Health	Factors		
	Age *	Household	Combination		Age	Household	Combination		Age	Household	Combination		Age	Household	Combination
HC1		**✓**		PC1	**✓**		**✓**	LC1				HT1			
HC2				PC2 (#)		**✓**	**✓**	LC2	**✓**		**✓**	HT2			
HC3	**✓**			PC3 (#)			**✓**	LC3	**✓**			HT3			
HC4				PC4				LC4 (#)	**✓**	**✓**	**✓**	HT4			
HC5				PC5				LC5	**✓**		**✓**				
HC6				PC6	**✓**		**✓**	LC6							
HC7				PC7	**✓**	**✓**	**✓**	LC7							
HC8								LC8	**✓**						
HC9								LC9	**✓**		**✓**				
HC10															
HC11															
HC12	**✓**														
HC13	**✓**		**✓**												
HC14															
HC15	**✓**		**✓**												
HC16	**✓**														
HC17 (#)															
HC18 (#)															
HC19 (#)	**✓**		**✓**												
HC20	**✓**														

* The willingness to use scores for the “household chores” category differed significantly between the younger group and the older group. # interaction effect.

## Data Availability

The data are included in the article; further inquiries can be directed to the corresponding author due to privacy concerns.

## Appendix A

We provide a detailed description of the survey items conducted by Research & Yu, as requested. The first page of the survey consists of a consent form for participation and collects basic demographic information from the respondents. It includes a statement assuring participants that their responses and personal data will be used solely for research purposes. This is conducted in compliance with Sections 33 (Protection of Confidentiality) and 34 (Duties of Statisticians) of the Statistics Act, ensuring that all survey responses remain anonymized for analysis. The subsequent pages are divided into two parts: Part A, titled “Survey on Willingness to Use Robot Tasks”, and Part B, “Survey on Preferences and Perceptions of Robots”. Each part is designed to gather information on respondents’ attitudes to various aspects of robot usage and preferences.

**First page of survey**

This survey is being conducted to study preferences for tasks of home robots.

Please answer the survey as if you have a home robot in your home or if you are considering a new home robot.

Your responses and your personal data will never be used for any purpose other than research, in accordance with Sections 33 (Protection of Confidentiality) and 34 (Duties of Statisticians) of the Statistics Act, and all surveys will be anonymized for analysis.

Do you agree to participate in this study?

□ I agree.

□ I do not agree → Stop survey

**Respondent background**

**1**	Age	□ 20~29 years old □ 30~39 years old □ 40~49 years old □ 50~59 years old
**2**	Gender	□ Male □ Female
**3**	Household type	□ SPH (Single-Person Household) □ MPH (Multi-Person Household)

A. **Survey on willingness to use robot tasks**
A.1. Please indicate the extent to which you would be willing to use a home robot for each task category below.

1	Tasks related to household chores	□	□	□	□	□
		Not at all willing to use	Not willing to use	Moderately willing to use	Highly willing to use	Extremely willing to use
2	Tasks related to personal care	□	□	□	□	□
		Not at all willing to use	Not willing to use	Moderately willing to use	Highly willing to use	Extremely willing to use
3	Tasks related to leisure/companion	□	□	□	□	□
		Not at all willing to use	Not willing to use	Moderately willing to use	Highly willing to use	Extremely willing to use
4	Tasks related to health	□	□	□	□	□
		Not at all willing to use	Not willing to use	Moderately willing to use	Highly willing to use	Extremely willing to use

A.2. Please indicate the extent to which you would be willing to use a home robot for each task in the ‘household chores’ category below.

1	Prepare meals	□	□	□	□	□
		Not at all willing to use	Not willing to use	Moderately willing to use	Highly willing to use	Extremely willing to use
2	Set table	□	□	□	□	□
		Not at all willing to use	Not willing to use	Moderately willing to use	Highly willing to use	Extremely willing to use
3	Grocery shop	□	□	□	□	□
		Not at all willing to use	Not willing to use	Moderately willing to use	Highly willing to use	Extremely willing to use
4	Wash dishes by hand	□	□	□	□	□
		Not at all willing to use	Not willing to use	Moderately willing to use	Highly willing to use	Extremely willing to use
5	Clean refrigerator	□	□	□	□	□
		Not at all willing to use	Not willing to use	Moderately willing to use	Highly willing to use	Extremely willing to use
6	Laundry	□	□	□	□	□
		Not at all willing to use	Not willing to use	Moderately willing to use	Highly willing to use	Extremely willing to use
7	Water plants	□	□	□	□	□
		Not at all willing to use	Not willing to use	Moderately willing to use	Highly willing to use	Extremely willing to use
8	Sort mail	□	□	□	□	□
		Not at all willing to use	Not willing to use	Moderately willing to use	Highly willing to use	Extremely willing to use
9	Use dishwasher	□	□	□	□	□
		Not at all willing to use	Not willing to use	Moderately willing to use	Highly willing to use	Extremely willing to use
10	Take out trash/recyclables	□	□	□	□	□
		Not at all willing to use	Not willing to use	Moderately willing to use	Highly willing to use	Extremely willing to use
11	Make bed/change sheets	□	□	□	□	□
		Not at all willing to use	Not willing to use	Moderately willing to use	Highly willing to use	Extremely willing to use
12	Change light bulbs	□	□	□	□	□
		Not at all willing to use	Not willing to use	Moderately willing to use	Highly willing to use	Extremely willing to use
13	Clean bedrooms	□	□	□	□	□
		Not at all willing to use	Not willing to use	Moderately willing to use	Highly willing to use	Extremely willing to use
14	Clean windows	□	□	□	□	□
		Not at all willing to use	Not willing to use	Moderately willing to use	Highly willing to use	Extremely willing to use
15	Clean floors	□	□	□	□	□
		Not at all willing to use	Not willing to use	Moderately willing to use	Highly willing to use	Extremely willing to use
16	Clean kitchen	□	□	□	□	□
		Not at all willing to use	Not willing to use	Moderately willing to use	Highly willing to use	Extremely willing to use
17	Walk with pet	□	□	□	□	□
		Not at all willing to use	Not willing to use	Moderately willing to use	Highly willing to use	Extremely willing to use
18	Feed pet	□	□	□	□	□
		Not at all willing to use	Not willing to use	Moderately willing to use	Highly willing to use	Extremely willing to use
19	Wash pet	□	□	□	□	□
		Not at all willing to use	Not willing to use	Moderately willing to use	Highly willing to use	Extremely willing to use
20	Manipulating objects	□	□	□	□	□
		Not at all willing to use	Not willing to use	Moderately willing to use	Highly willing to use	Extremely willing to use

A.3. Please indicate the extent to which you would be willing to use a home robot for each task in the ‘personal care’ category below.

1	Shave	□	□	□	□	□
		Not at all willing to use	Not willing to use	Moderately willing to use	Highly willing to use	Extremely willing to use
2	Wash/comb hair	□	□	□	□	□
		Not at all willing to use	Not willing to use	Moderately willing to use	Highly willing to use	Extremely willing to use
3	Bathe	□	□	□	□	□
		Not at all willing to use	Not willing to use	Moderately willing to use	Highly willing to use	Extremely willing to use
4	Eat/feed myself	□	□	□	□	□
		Not at all willing to use	Not willing to use	Moderately willing to use	Highly willing to use	Extremely willing to use
5	Get dressed	□	□	□	□	□
		Not at all willing to use	Not willing to use	Moderately willing to use	Highly willing to use	Extremely willing to use
6	Walk	□	□	□	□	□
		Not at all willing to use	Not willing to use	Moderately willing to use	Highly willing to use	Extremely willing to use
7	Brushing teeth	□	□	□	□	□
		Not at all willing to use	Not willing to use	Moderately willing to use	Highly willing to use	Extremely willing to use

A.4. Please indicate the extent to which you would be willing to use a home robot for each task in the ‘leisure/companion’ category below.

1	Entertain guests	□	□	□	□	□
		Not at all willing to use	Not willing to use	Moderately willing to use	Highly willing to use	Extremely willing to use
2	Being entertained	□	□	□	□	□
		Not at all willing to use	Not willing to use	Moderately willing to use	Highly willing to use	Extremely willing to use
3	Call family/friends	□	□	□	□	□
		Not at all willing to use	Not willing to use	Moderately willing to use	Highly willing to use	Extremely willing to use
4	Learn to use new technology	□	□	□	□	□
		Not at all willing to use	Not willing to use	Moderately willing to use	Highly willing to use	Extremely willing to use
5	Get information on hobbies	□	□	□	□	□
		Not at all willing to use	Not willing to use	Moderately willing to use	Highly willing to use	Extremely willing to use
6	Learn new skills	□	□	□	□	□
		Not at all willing to use	Not willing to use	Moderately willing to use	Highly willing to use	Extremely willing to use
7	Exercising together	□	□	□	□	□
		Not at all willing to use	Not willing to use	Moderately willing to use	Highly willing to use	Extremely willing to use
8	Reading stories	□	□	□	□	□
		Not at all willing to use	Not willing to use	Moderately willing to use	Highly willing to use	Extremely willing to use
9	Conversation	□	□	□	□	□
		Not at all willing to use	Not willing to use	Moderately willing to use	Highly willing to use	Extremely willing to use

A.5. Please indicate the extent to which you would be willing to use a home robot for each task in the ‘health’ category below.

1	Decision for medication	□	□	□	□	□
		Not at all willing to use	Not willing to use	Moderately willing to use	Highly willing to use	Extremely willing to use
2	Remind to take medicine	□	□	□	□	□
		Not at all willing to use	Not willing to use	Moderately willing to use	Highly willing to use	Extremely willing to use
3	Exercise	□	□	□	□	□
		Not at all willing to use	Not willing to use	Moderately willing to use	Highly willing to use	Extremely willing to use
4	Call doctors/911	□	□	□	□	□
		Not at all willing to use	Not willing to use	Moderately willing to use	Highly willing to use	Extremely willing to use

B. **Survey on preference and perceptions of robots**
B.1. Please indicate to what extent you prefer human or robotic assistance for the following task categories.

1	Tasks related to household chores	□ Entirely human	□ Human	□ Any of them	□ Robot	□ Entirely robotic
2	Tasks related to personal care	□ Entirely human	□ Human	□ Any of them	□ Robot	□ Entirely robotic
3	Tasks related to leisure/companion	□ Entirely human	□ Human	□ Any of them	□ Robot	□ Entirely robotic
4	Tasks related to health	□ Entirely human	□ Human	□ Any of them	□ Robot	□ Entirely robotic

B.2. The following questions ask about the reasons for your preference for a home robot and its status/role in your home. Please tick all that apply. For others, please write your own thoughts.

1	If you prefer a home robot, what is the reason for that?	□ Overcoming physical challenges □ Saving time (Rest or focus on other things while the robot does the work for you) □ Saving effort (Take over tedious, repetitive tasks) □ Confidence in capabilities (Result of the task is better than what a human would do) □ Social features (Talk/Game together) □ Others (_____________)
2	If you have a home robot, what is your expected/desired status/position in your home?	□ Tools (Appliances) □ Secretary/Butler □ Friend □ Family member □ Pet □ Others (_____________)

(end of survey)
